# *PITX3* promoter methylation is a prognostic biomarker for biochemical recurrence-free survival in prostate cancer patients after radical prostatectomy

**DOI:** 10.1186/s13148-016-0270-x

**Published:** 2016-09-26

**Authors:** Emily Eva Holmes, Diane Goltz, Verena Sailer, Maria Jung, Sebastian Meller, Barbara Uhl, Jörn Dietrich, Magda Röhler, Jörg Ellinger, Glen Kristiansen, Dimo Dietrich

**Affiliations:** 1 Institute of Pathology, University Hospital Bonn, Sigmund-Freud-Str. 25, 53127 Bonn, Germany; 2 Department of Pathology and Laboratory Medicine, Weill Cornell Medicine of Cornell University, New York, NY USA; 3 Englander Institute for Precision Medicine, Weill Cornell Medicine of Cornell University, New York, NY USA; 4 Department of Otolaryngology, Head and Neck Surgery, University Hospital Bonn, Bonn, Germany; 5 Department of Urology, University Hospital Bonn, Bonn, Germany

**Keywords:** *PITX3*, *PITX2*, Prostate cancer, DNA methylation, Prognostic biomarker

## Abstract

**Background:**

Molecular biomarkers that might help to distinguish between more aggressive and clinically insignificant prostate cancers (PCa) are still urgently needed. Aberrant DNA methylation as a common molecular alteration in PCa seems to be a promising source for such biomarkers. In this study, *PITX3* DNA methylation (*mPITX3*) and its potential role as a prognostic biomarker were investigated. Furthermore, m*PITX3* was analyzed in combination with the established PCa methylation biomarker *PITX2* (*mPITX2*).

**Methods:**

*mPITX3* and *mPITX2* were assessed by a quantitative real-time PCR and by means of the Infinium HumanMethylation450 BeadChip. BeadChip data were obtained from The Cancer Genome Atlas (TCGA) Research Network. DNA methylation differences between normal adjacent, benign hyperplastic, and carcinomatous prostate tissues were examined in the TCGA dataset as well as in prostatectomy specimens from the University Hospital Bonn. Retrospective analyses of biochemical recurrence (BCR) were conducted in a training cohort (*n* = 498) from the TCGA and an independent validation cohort (*n* = 300) from the University Hospital Bonn. All patients received radical prostatectomy.

**Results:**

In PCa tissue, *mPITX3* was increased significantly compared to normal and benign hyperplastic tissue. In univariate Cox proportional hazards analyses, *mPITX3* showed a significant prognostic value for BCR (training cohort: hazard ratio (HR) = 1.83 (95 % CI 1.07–3.11), *p* = 0.027; validation cohort: HR = 2.56 (95 % CI 1.44–4.54), *p* = 0.001). A combined evaluation with *PITX2* methylation further revealed that hypermethylation of a single *PITX* gene member (either *PITX2* or *PITX3*) identifies an intermediate risk group.

**Conclusions:**

*PITX3* DNA methylation alone and in combination with *PITX2* is a promising biomarker for the risk stratification of PCa patients and adds relevant prognostic information to common clinically implemented parameters. Further studies are required to determine whether the results are transferable to a biopsy-based patient cohort. Trial registration: Patients for this unregistered study were enrolled retrospectively.

## Background

Prostate cancer (PCa) is the most common cancer in men in the western hemisphere. In 2015, 220,800 new cases and 27,540 tumor-related deaths were predicted for the USA [1]. In the last couple of decades, prostate-specific antigen (PSA) screening has increased the number of early detected PCa [2]. However, the natural course of these tumors is highly variable. A majority of cases progresses slowly, remains years to decades in a clinically dormant state, and may be safely kept under active surveillance. Others develop fast and lead to locally aggressive growth and metastasis after short courses of disease. In the long run, these patients might benefit from a more radical treatment when diagnosed at a very early stage. Clinicopathological parameters, i.e., PSA values, tumor size, number of positive biopsies, and Gleason grading groups, as suggested by the International Society of Urological Pathologists (ISUP), guide the decision-making process when determining whether a patient may benefit from radical prostatectomy or can instead be closely monitored. However, in many cases, this approach has not proven satisfactory in that patients either suffered from overtreatment or experienced very early PSA relapses after surgery [3, 4]. Therefore, new prognostic tools are still urgently needed to distinguish between the aggressive and indolent subtypes of PCa.

As potential biomarkers, epigenetic modifications such as hyper- or hypomethylation of tumor-related genes have lately emerged as one of the key alterations in cancer development [5–7]. Aberrant patterns of methylation have aroused interest in the molecular subclassification of urologic tumors and might potentially serve as prognostic and predictive biomarkers in PCa [8, 9]. Furthermore, DNA is a highly robust cellular element that can be extracted reliably from different materials, e.g., fresh tissue, formalin-fixed paraffin-embedded tissue (FFPET), and body fluids [10, 11].

Methylation of the paired-like homeodomain transcription factor 2 (*PITX2*) has been successfully proven a powerful prognostic biomarker in several cancer entities such as lung cancer [12], hormone-receptor-negative breast cancer [13–16], and PCa [17–19]. PITX2 is initiated by Wnt/β-catenin and is involved in the control of cell proliferation [20]. PITX2 regulates the expression of the androgen receptor (AR) and insulin-like growth factor (IGF) receptor genes, leading to the regulation of signaling pathways involving AR and IGF during PCa progression [21].

The paired-like homeodomain transcription factor 3 or pituitary homeobox 3 (*PITX3*) is a transcription factor belonging to the same protein family as *PITX2* [22]. *PITX3* has been shown to be transiently expressed in the eye lens and skeletal muscle during embryogenesis [23, 24]. Very recently, it has been reported that exposure to environmental xenoestrogens may lead to neonatal DNA methylation re-programing effects in the prostate including dysregulation of *PITX3* methylation [25]. This may potentially foster carcinogenesis. Moreover, *PITX3* has previously been found to be aberrantly methylated in breast cancer patients [26].

These findings prompted us to investigate *PITX3* promoter methylation in PCa in a publically available dataset from The Cancer Genome Atlas (TCGA) [27] (training cohort) and an independent primary PCa patient cohort from the University Hospital Bonn (validation cohort).

## Results

### *PITX3* and *PITX2* promoter methylation in prostate tissues from TCGA training cohort

**Fig. 1:**
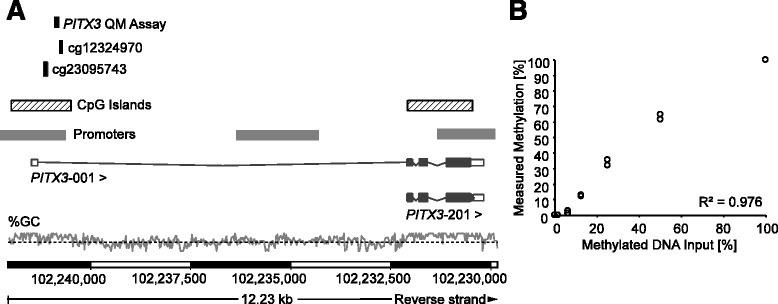
Genomic location, design, and validation of the *PITX3* QM Assay. **a** *PITX3* quantitative-methylation (QM) assay located on the reverse strand of chromosome 10. Both *PITX3* splice variants *PITX3-001* and *PITX3-201* are shown. The information was taken from Ensembl Homo sapiens version 82.38 (GRCh38.p3). The two beads of the Illumina Infinium HumanMethylation450 BeadChip (cg12324970 and cg23095743) used from the TCGA dataset are shown. The GC content (%) is shown with the *dashed line* indicating 50 % GC. **b** The QM real-time PCR assay was validated on a dilution series of bisulfite-converted artificially methylated and unmethylated DNA. Each sample was measured in duplicate

**Fig. 2:**
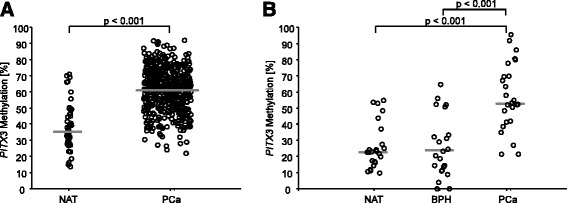
*PITX3* DNA methylation in prostatectomy specimens. Median methylation is indicated by the *gray line. PITX3* DNA methylation is significantly higher in prostate cancer (*PCa*) tissue compared to corresponding normal adjacent tissue (*NAT*) and benign prostatic hyperplasia (*BPH*). **a** NAT and PCa samples of the training cohort (TCGA). *p* values refer to Wilcoxon-Mann-Whitney test. **b** NAT, BPH, and PCa samples from a test study comprising 71 patient samples. Each sample was measured in triplicate. *p* values refer to Kruskal-Wallis test

**Fig. 3:**
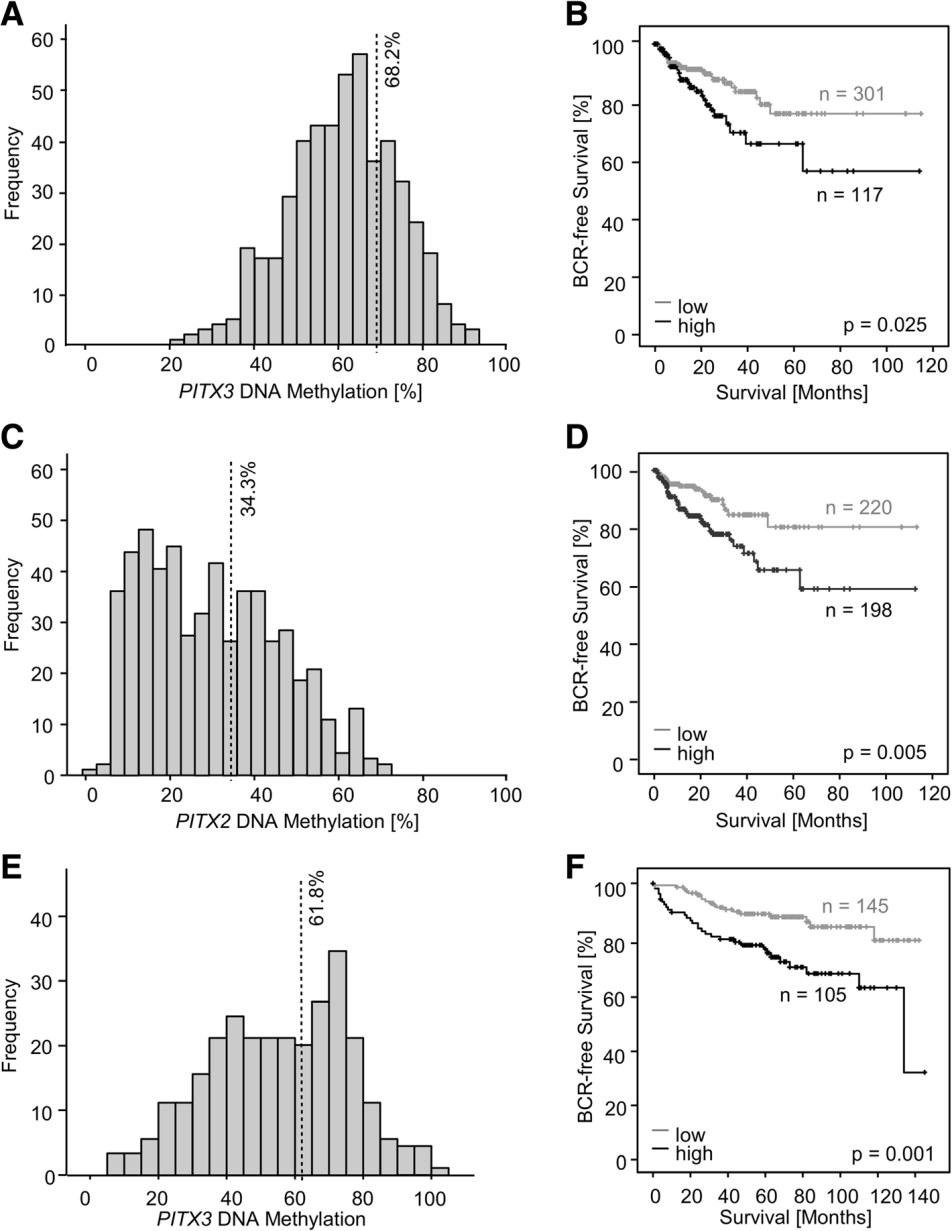
Frequency and prognostic value of *mPITX3* and *mPITX2* in the training (*n* = 498) and validation (*n* = 300) cohorts. *PITX3* and *PITX2* DNA methylation was analyzed in prostate carcinoma patients from two cohorts. Methylation frequencies (**a**, **c**, and **e**) and Kaplan-Meier analyses of BCR-free survival in patients stratified according to dichotomized *mPITX3* and *mPITX2* levels are shown (**b**, **d**, and **f**). **a** *mPITX3* analysis in the training cohort revealed a symmetric, bell-shaped distribution covering a broad spectrum of values (22–92 %). An optimal cutoff was elaborated by an iterative approach (68.2 %) stratifying patients into *mPITX3* hyper- (*mPITX3*_high_) and hypomethylated (*mPITX3*_low_) cases. **b** Patient survival in the training cohort according to *mPITX3*_low_ and *mPITX3*_high_ status. Patients with m*PITX3*_low_ tumors show a better prognosis. Approximate mean BCR-free survival: 93 months (m*PITX3*_low_, 95 % CI 85–100 months, *n* = 301) and 76 months (*mPITX3*_high_, 95 % CI 63–90 months, *n* = 117; LR = 5.05; *p* = 0.025), respectively. **c** *mPITX2* analysis in the training cohort revealed an uneven distribution covering an altogether lower spectrum of values than *mPITX3* (5–79 %). An optimal cutoff was elaborated by an iterative approach (34.3 %) stratifying patients into *mPITX2* hyper- (*mPITX2*_high_) and hypomethylated (*mPITX2*_low_) cases. **d** Patient survival in the validation cohort according to *mPITX2*_low_ and *mPITX2*_high_ status. Patients with m*PITX2*_low_ tumors show a better prognosis. Approximate mean BCR-free survival: 96 months (m*PITX2*_low_, 95 % CI 88–105 months, *n* = 220) and 78 months (*mPITX2*_high_, 95 % CI 67–89 months, *n* = 198; LR = 7.95; *p* = 0.005), respectively. **e** *mPITX3* analysis in the validation cohort revealed a flattened, bell-shaped distribution covering (5–100 %). An optimal cutoff was elaborated by an iterative approach (61.8 %) stratifying patients into *mPITX3* hyper- (*mPITX3*_high_) and hypomethylated (*mPITX3*_low_) cases. **f** Patient survival in the validation cohort according to *mPITX3*_low_ and *mPITX3*_high_ status. Patients with *mPITX2*_low_ tumors show a better prognosis. Approximate mean BCR-free survival: 125 months (*mPITX3*_low_, 95 % CI 118–132 months, *n* = 145) and 103 months (*mPITX3*_high_, 95 % CI 91–115 months, *n* = 105; LR = 11.17; *p* = 0.001), respectively. Patient survival in the validation cohort according to *mPITX2*_low_ and *mPITX2*_high_ status is reported elsewhere [30]

For the analysis of *PITX3* promoter methylation (*mPITX3*) in the training cohort, results obtained from two Illumina Infinium HumanMethylation450 BeadChip beads from the TCGA dataset (cg12324970 and cg23095743) were used. Both beads were located within the CpG island of *PITX3* (Fig. 1a). Firstly, PCa (*n* = 498) and normal adjacent tissue (NAT, *n* = 50) samples from the training cohort were analyzed with respect to *mPITX3*. Patient samples showed a significantly lower level of *mPITX3* in NAT compared to PCa samples (*p* < 0.001, Fig. 2a). A histogram of *mPITX3* showed a bell curve with a minor depression at ≈68 % (Fig. 3a). *mPITX3* levels as a continuous variable were related to prognostic clinicopathological variables and were found to be significantly correlated with the ISUP Gleason grading group (*ρ* = 0.112; *p* = 0.012), pathologic tumor (pT) category (*ρ* = 0.123; *p* = 0.006), presurgical PSA (*ρ* = 0.134; *p* = 0.003), and the AR activity score (*ρ* = 0.154; *p* = 0.005) as obtained from TCGA [27] in the training cohort. In order to analyze the suitability of *mPITX3* for the stratification of patients at risk for biochemical recurrence (BCR), *mPITX3* was dichotomized by an optimized cutoff (*mPITX3*_low_ < 68.2 % ≤ *mPITX3*_high_; Table 1) which was identified by an iterative approach. In the training cohort, *mPITX3*_high_ was significantly associated with BCR in the univariate Cox proportional hazards model (hazard ratio (HR) = 1.83 (95 % CI 1.07–3.11); *p* = 0.027; (Table 2)) and the Kaplan-Meier analysis (likelihood ratio (LR) = 5.05; *p* = 0.025, Fig. 3b).

**Table 1:** Associations of *PITX3* DNA methylation (*mPITX3*) with clinicopathological parameters of PCa patients from the training (*n* = 498) and validation cohort (*n* = 300)

	Training cohort					Validation cohort				
	Patients (*n*)	Median *mPITX3* (%)	*mPITX3*_low_	*mPITX3*_high_	*p* value	Patient (*n*)	Median *mPITX3* (%)	*PITX3*_low_	*mPITX3*_high_	*p* value
All patients	498 (100 %)	62.0				300 (100 %)	57.9			
Mean/median follow-up (months)	22/16					66/63				
Age (years)					0.021^a^					0.011^a^
≤60	224 (45.0 %)	60.2	166 (73.8 %)	58 (25.8 %)		71 (23.7 %)	50.0	48 (64.0 %)	23 (30.7 %)	
>60	274 (55.0 %)	62.9	184 (66.9 %)	90 (32.7 %)		219 (73.0 %)	61.0	115 (51.3 %)	104 (46.4 %)	
Unknown	0 (0.0 %)					10 (3.3 %)				
T category					0.017^a^					<0.001^a^
pT1/2	188 (37.8 %)	59.6	147 (78.2 %)	41 (21.8 %)		198 (66.0 %)	53.4	128 (62.4 %)	70 (34.1 %)	
pT3/4	293 (58.8 %)	63.3	189 (64.1 %)	104 (35.3 %)		88 (29.3 %)	69.0	32 (35.6 %)	56 (62.2 %)	
Unknown	17 (3.4 %)					14 (4.7 %)				
ISUP Gleason grading group					0.035^b^					0.029^b^
1 (<7)	45 (9 %)	61.6	33 (73.3 %)	12 (26.7 %)		155 (51.7 %)	53.1	99 (60.7 %)	56 (34.4 %)	
2 (3 + 4)	147 (29.5 %)	59.5	118 (79.2 %)	29 (19.5 %)		53 (17.7 %)	58.5	29 (54.7 %)	24 (45.2 %)	
3 (4 + 3)	101 (20.3 %)	62.1	67 (66.3 %)	34 (33.7 %)		23 (7.7 %)	69.0	10 (43.5 %)	13 (56.5 %)	
4 (=8)	64 (12.9 %)	61.0	34 (67.2 %)	21 (32.8 %)		34 (11.3 %)	61.1	17 (48.6 %)	17 (48.6 %)	
5 (>8)	141 (28.3 %)	64.1	89 (63.1 %)	52 (36.9 %)		15 (5.0 %)	66.4	3 (18.8 %)	12 (75.0 %)	
Unknown	0 (0.0 %)					20 (6.7 %)				
Surgical margin					0.19^a^					0.62^a^
R0	318 (63.9 %)	60.2	227 (71.4 %)	89 (28.0 %)		198 (66.0 %)	55.3	117 (49.1 %)	74 (37.4 %)	
R1	152 (30.5 %)	63.5	103 (67.8 %)	49 (32.2 %)		96 (32.0 %)	62.7	44 (45.8 %)	50 (52.1 %)	
Unknown	28 (5.6 %)					6 (2.0 %)				
Nodal status					0.75^a^					0.66^a^
pN0	349 (70.1 %)	61.7	243 (69.8 %)	103 (29.6 %)		279 (93.0 %)	57.5	152 (54.5 %)	117 (41.9 %)	
pN1	79 (15.8 %)	61.6	55 (69.6 %)	24 (30.4 %)		17 (5.7 %)	61.7	9 (52.9 %)	8 (47.1 %)	
Unknown	70 (14.1 %)					4 (1.3 %)				
Pre-surgical PSA (ng/ml)					0.051^b^					0.089^b^
0–4	53 (10.6 %)	60.0	39 (73.4 %)	14 (26.4 %)		24 (8.70 %)	49.4	19 (70.4 %)	5 (18.5 %)	
4–10	286 (57.5 %)	60.5	210 (73.4 %)	76 (26.6 %)		169 (56.3 %)	58.3	95 (54.9 %)	74 (42.8 %)	
>10	156 (31.3 %)	64.0	98 (62.0 %)	58 (36.7 %)		84 (28.0 %)	61.0	43 (49.4 %)	41 (47.1 %)	
Unknown	3 (0.6 %)					23 (7.7 %)				
ERG fusion^c^					0.58^a^					0.15^a^
Negative	178 (35.8 %)	61.7	125 (70.2 %)	53 (29.8 %)		164 (54.7 %)	68.9	65 (44.5 %)	74 (50.7 %)	
Positive	152 (30.5 %)	62.9	106 (69.7 %)	46 (30.3 %)		56 (18.7 %)	65.7	27 (41.5 %)	35 (53.8 %)	
Unknown	168 (33.7 %)					80 (26.7 %)				
AR score					0.35^a^					0.32^a^
Negative	246 (49.4 %)	61.4	124 (74.7 %)	42 (25.3 %)		83 (27.7 %)	68.95	40 (48.2 %)	40 (48.2 %)	
Positive	84 (16.9 %)	64.5	109 (35.3 %)	58 (34.7 %)		81 (27.0 %)	68.5	46 (56.8 %)	34 (42.0 %)	
Unknown	186 (33.7 %)					136 (45.3 %)				

m*PITX3* was dichotomized by the respective optimized cutoff into m*PITX3*_low_ vs. m*PITX3*_high_

^a^Wilcoxon-Mann-Whitney test

^b^Kruskal-Wallis test

^c^Training cohort: ERG fusion as adopted from The Cancer Genome Atlas Research Network (2015) [27]; validation cohort: nuclear ERG protein expression

**Table 2:** Univariate Cox proportional hazard analysis of BCR-free survival in the training and validation cohort including PCa patients treated by radical prostatectomy

	Training cohort			Validation cohort		
Clinicopathological parameters/biomarker	*n*	Hazard ratio (95 % CI)	*p* value	*n*	Hazard ratio (95 % CI)	*p* value
Age	411	1.02 (0.98–1.06)	0.39	259	1.01 (0.96–1.06)	0.70
Tumor stage (pT3 and pT4 vs. pT2 and pT1)	346	4.25 (2.37–7.61)	<0.001	260	2.07 (1.30–3.30)	0.001
ISUP Gleason grading group	411	1.69 (1.34–2.13)	<0.001	252	1.99 (1.63–2.42)	<0.001
Surgical margin (R1 vs. R0)	389	1.49 (0.87–2.56)	0.15	258	1.00 (0.98–1.02)	0.84
Nodal status (pN1 vs. pN0)	357	1.84 (1.00–3.36)	0.048	259	1.09 (0.50–2.41)	0.82
Preoperative PSA level	409	1.04 (1.02–1.05)	<0.001	250	1.01 (1.00–1.02)	0.11
AR activity score (positive vs. negative)	271	0.74 (0.32–1.71)	0.49	NA	NA	NA
AR protein expression (AR high vs. AR low)	NA	NA	NA	143	0.82 (0.40–1.70)	0.60
ERG^a^ (*ERG*-fusion positive vs. *ERG*-fusion negative)	271	0.80 (0.40–1.57)	0.51	182	0.78 (0.40–1.51)	0.46
*mPITX3* (optimized cutoff, *mPITX3*_high_ vs. *mPITX3*_low_)	411	1.83 (1.07–3.11)	0.027	250	2.56 (1.44–4.54)	0.001

Only patients with available follow-up were included into this analysis

*NA* not analyzed

^a^Training cohort: *ERG*-fusion as adopted from The Cancer Genome Atlas Research Network (2015) [27]; validation cohort: nuclear ERG protein expression as surrogate marker for *ERG*-translocation

*PITX3* carries a homeodomain which is highly homologous with *PITX2* [28]. In a second step, *PITX2* promoter methylation (*mPITX2*) was therefore analyzed in an equivalent manner. A *PITX2* quantitative methylation-specific real-time polymerase chain reaction (PCR) (qMSP) assay has been established and validated in a previous study using other patient material [29]. Three beads from the HumanMethylation450 BeadChip which are located in close proximity of the established qMSP assay were selected. *mPITX2* showed a rather asymmetrical distribution (Fig. 3c). Associations of *mPITX2* levels with clinicopathological variables in the training cohort are shown in Table 3. In brief, *mPITX2* levels correlated with age, T category, ISUP Gleason grading group, surgical margin, and *ETS*-related gene (*ERG*) fusion status. Dichotomization by an optimized cutoff (*mPITX2*_low_ < 34.3 % ≤ *mPITX2*_high_) revealed a significant prognostic value. In the training cohort, *mPITX2*_high_ was significantly associated with BCR in the univariate Cox proportional hazards model (HR = 2.20 (95 % CI 1.25–3.87); *p* = 0.006) and the Kaplan-Meier analysis (LR = 7.95; *p* = 0.005, Fig. 3d).

**Table 3:** Associations of *PITX2* DNA methylation (*mPITX2*) with clinicopathological parameters of PCa patients from the training cohort (*n* = 498)

	Patients (*n*)	Median *mPITX2* (%)	*mPITX2*_low_	*mPITX2*_high_	*p* value
All patients	498 (100 %)	32.9			
Mean/median follow-up (months)	22/16				
Age (years)					0.001^a^
≤60	224 (45.0 %)	31.0	128 (57.1 %)	96 (42.9 %)	
>60	274 (55.0 %)	35.2	135 (49.1 %)	140 (50.9 %)	
Unknown	0 (0.0 %)				
T category					0.043^a^
pT1/2	188 (37.8 %)	25.7	137 (70.3 %)	58 (29.7 %)	
pT3/4	293 (58.8 %)	37.5	126 (41.4 %)	178 (58.6 %)	
Unknown	17 (3.4 %)				
ISUP Gleason grading group					<0.001^b^
1 (<7)	45 (9 %)	28.0	31 (68.9 %)	14 (31.1 %)	
2 (3 + 4)	147 (29.5 %)	28.6	95 (64.2 %)	53 (35.8 %)	
3 (4 + 3)	101 (20.3 %)	31.5	55 (54.5 %)	46 (45.5 %)	
4 (=8)	64 (12.9 %)	34.1	33 (51.6 %)	31 (48.4 %)	
5 (>8)	141 (28.3 %)	41.0	49 (34.8 %)	92 (65.2 %)	
Unknown	0 (0.0 %)				
Surgical margin					<0.001^a^
R0	318 (63.9 %)	30.8	180 (56.8 %)	137 (43.2 %)	
R1	152 (30.5 %)	37.0	68 (44.7 %)	84 (55.3 %)	
Unknown	28 (5.6 %)				
Nodal status					0.19^a^
pN0	349 (70.1 %)	32.3	184 (53.0 %)	163 (47.0 %)	
pN1	79 (15.8 %)	36.6	32 (40.5 %)	47 (49.5 %)	
Unknown	70 (14.1 %)				
Pre-surgical PSA (ng/ml)					0.36^b^
0–4	53 (10.6 %)	29.5	31 (58.5 %)	122 (41.5 %)	
4–10	286 (57.5 %)	32.9	150 (52.4 %)	136 (47.6 %)	
>10	156 (31.3 %)	34.3	79 (50.3 %)	78 (49.7 %)	
Unknown	3 (0.6 %)				
ERG fusion^c^					<0.001^a^
Negative	178 (35.8 %)	28.0	122 (62.9 %)	66 (37.1 %)	
Positive	152 (30.5 %)	37.2	69 (45.4 %)	83 (54.6 %)	
Unknown	168 (33.7 %)				
AR score					0.15^a^
Negative	246 (49.4 %)	30.6	140 (56.9 %)	106 (43.1 %)	
Positive	84 (16.9 %)	34.6	41 (48.8 %)	43 (51.2 %)	
Unknown	186 (33.7 %)				

*mPITX2* in the validation cohort is described elsewhere [30]. *mPITX2* was dichotomized by the respective optimized cutoff into *mPITX2*_low_ vs. *mPITX2*_high_

^a^Wilcoxon-Mann-Whitney test

^b^Kruskal-Wallis test

^c^ERG fusion status as adopted from The Cancer Genome Atlas Research Network (2015) [27]

**Fig. 4:**
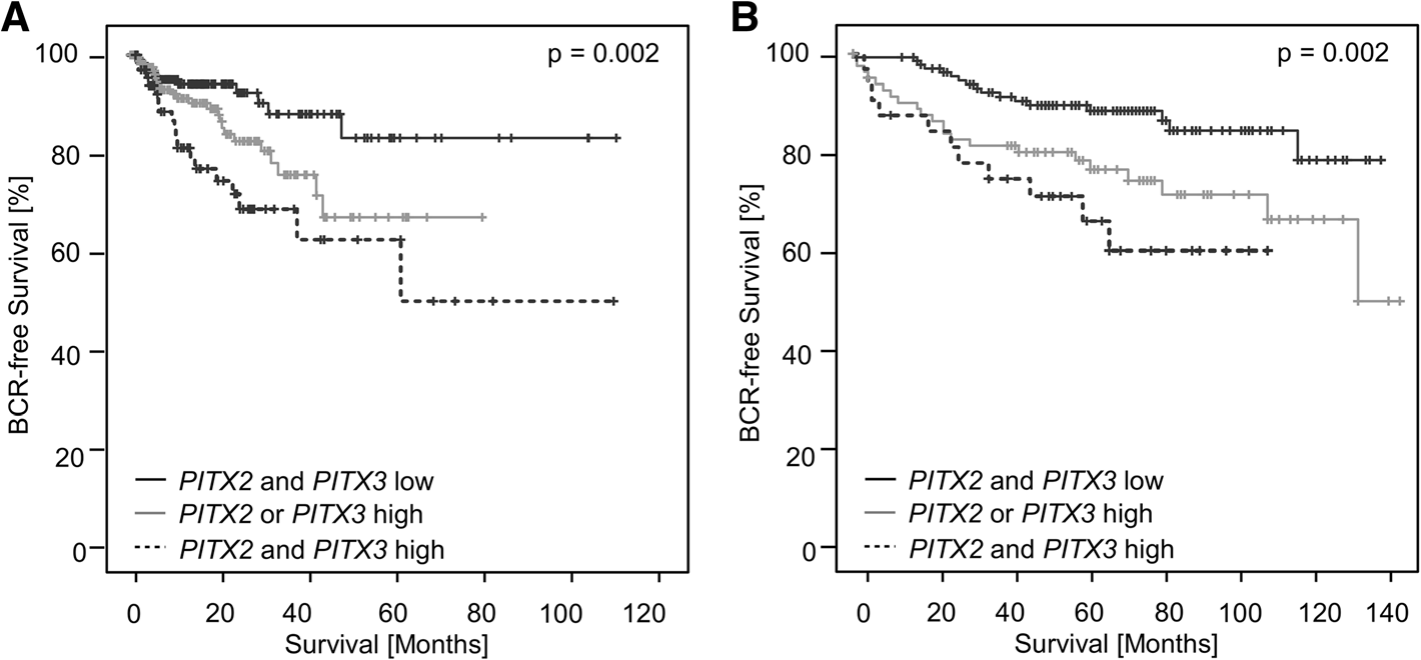
Survival according to combined *mPITX3* and *mPITX2* status. Kaplan-Meier analysis of BCR-free survival in prostate cancer patients stratified according to *PITX3* and *PITX2* DNA methylation status. Training cohort (*n* = 498, **a**): After a homogenous dropout within the first months after prostatectomy in all three groups, patients with low methylation values in *PITX2* and *PITX3* genes show the lowest number of BCR events (*n* = 182). Patients with high methylation in *PITX2* and *PITX3* genes present with the highest rate of BCR events (*n* = 67). Intermediate numbers of BCR events are observed in patients with low methylation in one *PITX* gene member and high methylation in the other *PITX* gene member (*n* = 169). Validation cohort (*n* = 300, **b**): Patients with low methylation values in *PITX2* and *PITX3* genes show the lowest number of BCR events (*n* = 136). Patients with high methylation in *PITX2* and *PITX3* genes present with the earliest BCR events (*n* = 32). Patients with low methylation in one *PITX* gene member and high methylation in the other *PITX* gene member (*n* = 82) show the highest number of BCR events, however, more protracted than patients with high methylation in both *PITX* genes

Since both parameters showed excellent prognostic performance, the combination of *mPITX2* and *mPITX3* was tested in the TCGA collective. Here, *mPITX2*_low_ and *mPITX3*_low_ cases showed significantly longer BRC-free survival compared to patients with *mPITX2*_high_ and/or *mPITX3*_high_ (LR = 12.70, *p* = 0.002; Fig. 4a).

### Analytical assay design and performance of the *mPITX3* real-time PCR

Following the analysis of the training cohort, a *PITX3* quantitative methylation (QM) assay was designed within the CpG island upstream of the *PITX3* gene in the same region as the beads selected from the HumanMethylation450 BeadChip analyzed by TCGA Research Network (Fig. 1a). In contrast to the established qMSP used to quantify *PITX2* methylation as described earlier [29], QM assay refers to an assay which is based on two primers which do not cover any CpG sites and therefore amplify unmethylated as well as methylated DNA. This assay contains two detection probes: One detection probe specifically binds to unmethylated DNA while the other probe specifically and competitively binds to methylated DNA. The assay performance was validated using a dilution series of bisulfite-converted artificially methylated and unmethylated DNA. The assay allowed for an accurate quantification of *mPITX3* within the whole spectrum from 0 to 100 % methylation (*r*^2^ = 0.98, Fig. 1b).

### *PITX3* promoter methylation in prostate tissues in the test study

In order to avoid artifacts which might result from a genome-wide methylation testing approach as used by the TCGA, the aforementioned findings from the TCGA cohort were confirmed in a small test study comprised of 71 samples from 25 prostatectomy specimens. *mPITX3* levels were significantly lower in NAT and samples with benign prostatic hyperplasia (BPH) compared to PCa samples (*p* < 0.001, Fig. 2b). No difference of *mPITX3* was detected in BPH compared to NAT samples.

### *PITX3* promoter methylation in prostate tissues from the validation cohort

In a validation cohort of 300 patients with clinical follow-up, *mPITX3* significantly correlated with the ISUP Gleason grading group (*ρ* = 0.193; *p* = 0.001), pT (*ρ* = 0.278; *p* < 0.001), and pre-surgical PSA (*ρ* = 0.143; *p* = 0.017). Associations of *mPITX2* with clinicopathologic parameters in the validation cohort have been described elsewhere [30]. In a histogram, the distribution of *mPITX3* resembled a flattened bell-shaped curve dichotomized by an optimized cutoff (m*PITX3*_low_ < 61.8 % ≤ m*PITX3*_high;_ Fig. 3e). In concordance with the training cohort, *mPITX3*_high_ was significantly associated with early BCR using an optimized cutoff (HR = 2.56 (95 % CI 1.44–4.54); *p* = 0.001, Table 2). This result was further confirmed by Kaplan-Meier analysis (LR = 11.17; *p* = 0.001; Fig. 3f). Additionally, *mPITX3* was significantly associated with BCR in the univariate Cox proportional hazards model when analyzed as continuous variable without cutoff-based dichotomization (HR = 1.02 (95 % CI 1.00–1.03), *p* = 0.025).

Since combined *mPITX2* and *mPITX3* revealed significant additive prognostic information in the training cohort, the combination of *mPITX2* and *mPITX3* was also tested in the validation cohort. According to the results obtained from the training cohort, *mPITX2*_low_ and *mPITX3*_low_ cases showed significantly longer BRC-free survival compared to patients with *mPITX2*_high_ and/or *mPITX3*_high_ (LR = 12.14, *p* = 0.002; Fig. 4b).

## Discussion

In this study, *PITX3* was shown to be aberrantly methylated in prostate carcinomas. *PITX3* was hypermethylated in PCa compared to normal adjacent prostate tissue in the training cohort and compared to both normal and benign prostatic hyperplasia in the test study. These findings are in line with previous reports on *PITX3* methylation in breast carcinoma [26].

Furthermore, carcinomatous *PITX3* hypermethylation was significantly associated with established clinicopathologic parameters characteristic of PCa. In detail, high ISUP Gleason grading group, advanced tumor stages, and high preoperative PSA values were related to high *PITX3* methylation in both cohorts. In addition, *PITX3* methylation correlated with a molecular AR activity score as obtained from TCGA Research Network [27], which was only available for the training cohort. An association with the ERG fusion protein or ERG protein expression could not be determined. Recently, dysregulation of *PITX3* methylation has been linked to the environmental burden of xenoestrogens [25]. In this respect, *PITX3* methylation may have an exceptional position among prognostic biomarkers. Of note, *PITX3* methylation served as a prognostic biomarker for BCR in both the training and validation cohort of radical prostatectomy patients. In Kaplan-Meier analysis, high *PITX3* methylation defined by an optimized cutoff for both patient groups was associated with a shorter BCR-free survival in the training and validation cohort. As a limiting condition, however, the follow-up period was shorter in the training cohort compared to the validation cohort, and the training cohort comprised significantly more high-grade carcinomas with an advanced stage and associated with earlier BCR. In the validation cohort, *PITX3* methylation succeeded as a prognostic factor dichotomized by an optimized cutoff and as a continuous variable in the univariate Cox proportional hazards analysis. In consideration of the fact that several recent studies have reported on a striking prognostic power of gene methylation of *PITX2* [17, 19], a close relative of *PITX3*, a combined analysis of *PITX2* and *PITX3* promoter methylation, was performed. Thereby, we intended to investigate possible interactions to compensate for gene methylation in either *PITX* member. Combined analysis of *PITX2* and *PITX3* promoter methylation revealed that low methylation in both genes was associated with favorable courses of disease in each cohort. Vice versa, patients with hypermethylated *PITX2* and *PITX3* promoters presented with the shortest BCR-free survival intervals after radical prostatectomy. Intermediate BRC-free survival intervals were observed in patients with low gene methylation in one *PITX* member and high methylation in the other *PITX* members. In respect thereof, we conclude that the analysis of *PITX3* gene methylation adds to the prognostic information obtained from *PITX2* analysis, suggesting that, in contrast to their overlapping functions in human development, they play a distinct role in the genesis and progression of PCa. This issue further needs to be confirmed in larger studies in which patient numbers allow for multivariate analysis. Furthermore, the prognostic value should be analyzed with regard to more clinically relevant endpoints, i.e., prostate cancer-specific survival, which unfortunately was not available for the present analyses.

The present study indicates that *PITX3* promoter methylation may be of great value for the tailoring of individual therapies and risk stratification. Even though PSA screening has led to a reduction of cases with advanced disease and disease-specific mortality, low-risk PCa rarely causes symptoms or affects survival if left untreated. Nevertheless, most men diagnosed with low-risk PCa in the USA receive up-front treatment, including prostatectomy or radiotherapy [31]. Hence, the early detection of low-risk PCa may lead to overdiagnosis resulting in overtreatment of patients with potential unnecessary side effects such as urinary dysfunction or impotence [3, 4]. The present study combines the analysis of *PITX3* promoter methylation in two independent cohorts and by two different molecular assays; however, further studies are warranted to scrutinize the potential of *PITX3* methylation as a biomarker prior to radical prostatectomy. Therefore, the assay’s prognostic power needs to be evaluated in biopsies from PCa patients included in an active surveillance protocol.

## Conclusions

In summary, *PITX3* DNA methylation is a promising biomarker for the risk stratification of PCa patients and adds relevant prognostic information to the common clinically implemented parameters. The prognostic power of *PITX3* DNA methylation was validated in two independent radical prostatectomy cohorts. Adjunct to the analysis of *PITX2* promoter methylation, hypermethylation of *PITX3* provided supplemental information on the course of disease, indicating adverse patient outcome. This implies a distinct function of the *PITX*3 gene in the development of PCa. However, the establishment of *PITX3* as a clinical prognostic marker needs to be established in further studies reappraising its transferability to biopsy-based patient cohorts.

## Methods

### Patients and clinical endpoint

#### Test study

A set of 71 FFPE prostate tissue samples from 25 PCa patients who underwent therapy at the University Hospital of Bonn in 2011 were included. The samples included 25 PCa, 24 NAT, and 22 BPH specimens.

#### Patient training cohort

A patient cohort comprised of 498 patients from the TCGA Research Network. Two Illumina Infinium HumanMethylation450 BeadChip beads (cg12324970 and cg23095743) were used to calculate relative methylation levels of *PITX3* by the formula 100 %*bead_M/(bead_M + bead_U). The average value of the ratios of the beads cg12324970 and cg23095743 was calculated. BCR-free survival was considered as the primary endpoint of the study. For *PITX2*, three Illumina Infinium HumanMethylation450 BeadChip beads (cg10391633, cg01616926, and cg19134945) were analyzed, accordingly.

#### Patient validation cohort

A patient cohort comprised of 300 patients with histologically confirmed PCa who underwent radical prostatectomy at the University Hospital Bonn between 1998 and 2008. BCR-free survival was considered the primary endpoint of the study and was determined as elevation PSA levels above 0.2 ng/ml.

### Sample preparation

For the analysis of *PITX3* methylation, the FFPET samples were processed according to the InnuCONVERT Bisulfite All-In-One Kit (Analytik Jena, Germany) as previously published [10]. To validate the assay performance, a serial dilution of bisulfite-converted artificially methylated DNA (CpGenome™ Universal Methylated DNA; Merck Millipore, Darmstadt, Germany) and unmethylated DNA from human sperm (NW Andrology & Cryobank Inc., Spokane, WA, USA) was used. As a calibrator sample DNA, a 1:1 mixture of bisulfite-converted unmethylated and artificially methylated DNA was used.

### *mPITX2* and *mPITX3* quantitative real-time PCR

The DNA methylation of *PITX2* and *PITX3* was determined by means of qMSP and QM PCR assays, respectively. The *PITX2* qMSP assay has been described earlier in detail [29]. Table 4 lists the primers and probes used for the QM *PITX3* assay. Each sample was measured in triplicate with an input of 25 ng bisulfite-converted DNA per reaction. The *PITX3* QM assay was performed using an AB 7500 Fast Real-Time PCR System (Life Technologies Corporation, Carlsbad, CA, USA), and the following temperature profile was used: 15 min at 95 °C (first denaturation), followed by 45 cycles of 95 °C for 15 s, 60 °C for 2 s, and 55 °C for 60 s. The thresholds and baselines for analysis were set as follows: 0.02 (threshold) and 3–22 (baseline) for the methylated and unmethylated probe. m*PITX3* was calculated using the ΔΔCT method:

$$ \Delta \mathrm{C}\mathrm{T} = \varDelta {\mathrm{CT}}_{PITX3\mathit{\hbox{-}}\mathrm{P}\hbox{-} \mathrm{U}} - \varDelta {\mathrm{CT}}_{PITX3\hbox{-} \mathrm{P}\hbox{-} \mathrm{M}},\ \varDelta \varDelta \mathrm{C}\mathrm{T} = \varDelta {\mathrm{CT}}_{\mathrm{sample}}-\varDelta {\mathrm{CT}}_{\mathrm{calibrator}},\ \mathrm{m} PITX 3 = 100/\left(1+2\hat{\mkern6mu} \left(\varDelta \varDelta CT\right)\right). $$

**Table 4:** Primer and probe sequences of the quantitative methylation (QM) real-time *PITX3* PCR

Primer/probe name	Primer/probe sequence
*PITX3*-F	5′-CTCTCACAACACAACTCCTATTC-3′
*PITX3*-R	5′-TTTAGGTTTAGATTTTTGGGGTT-3′
*PITX3*-P-M	5′-VIC-CGACCAAACGCACCCCG-BHQ-2-3′
*PITX3*-P-U	5′-FAM-ATACAACCAAACACACCCCAACTCC-BHQ-1-3′

### Immunohistochemistry

Immunohistochemical staining of ERG and AR was conducted at the Institute of Pathology in Bonn. Staining of the sections was performed using the LabVision Autostainer 480S system (Thermo Scientific, Waltham, MA, USA) along with the Thermo Scientific Reagents and the N-Histofine® DAB-3S detection kit. The AR staining was performed as previously described [32]. For the ERG staining, the following antibody and dilution was used: clone EPR3864 (Biologo, Kronshagen, Germany; 1:100).

### Statistical analyses

The statistical analyses were performed using SPSS, version 22 (SPSS Inc., Chicago, IL). The relationship between input DNA methylation and measured DNA methylation was assessed by linear regression. Statements regarding potential correlations of specific histology findings were made using the Spearman’s rank correlation coefficient (*ρ*). BCR-free survival analyses were conducted by Kaplan-Meier and univariate Cox proportional hazards regression analyses. Kaplan-Meier analysis was conducted using the log-rank test and likelihood ratios (LR). *p* values lower than 0.05 were considered significant. For the comparison of independent groups, Wilcoxon-Mann-Whitney test (for two groups) and the Kruskal-Wallis test (for more than two groups) were applied.

## Abbreviations

AR: Androgen receptor
BCR: Biochemical recurrence
BPH: Benign prostatic hyperplasia
CT: Cycle threshold
ERG: ETS-related gene
FFPET: Formalin Fixed Paraffin Embedded Tissue
HR: Hazard ratio
ISUP: International Society of Urological Pathology
LR: Likelihood ratio
*mPITX2*: Methylated *PITX2*
*mPITX3*: Methylated *PITX3*
NAT: Normal adjacent tissue
PCa: Prostate cancer
PCR: Polymerase chain reaction
PITX2: Paired-like homeodomain transcription factor 2
PITX3: Paired-like homeodomain transcription factor 3
PSA: Prostate-specific antigen
QM: Quantitative methylation
qMSP: Quantitative methylation-specific PCR
TCGA: The Cancer Genome Atlas
